# Modulating the Gut Microbiome as a Therapeutic Approach in Multiple Sclerosis: Implications for Gut‐Brain Interactions and Immune Pathways: A Narrative Review

**DOI:** 10.1002/brb3.71254

**Published:** 2026-02-05

**Authors:** Husna Irfan Thalib, Nuha Fatima, Faaleha Heba Fakruddin, Hosna Hamidullah Ali, Sariya Khan, Mohammed Talha Mohammed Zubair, Mable Pereira, Fatma E. Sayed Hassan

**Affiliations:** ^1^ General Medicine Practice Program Batterjee Medical College Jeddah Saudi Arabia; ^2^ School of Medicine Lincoln American University Georgetown Guyana; ^3^ Medical Physiology Department, Kasr Alainy, Faculty of Medicine Cairo University Giza Egypt; ^4^ Department of Physiology, General Medicine Practice Program Batterjee Medical College Jeddah Saudi Arabia

**Keywords:** fecal microbiota transplantation, gut microbiome, multiple sclerosis, neuroinflammation, probiotics

## Abstract

**Purpose:**

Multiple sclerosis (MS) is a chronic autoimmune disease of the central nervous system characterized by progressive disability. Emerging evidence has implicated gut microbiome dysbiosis, characterized by decreased short‐chain fatty acids (SCFAs)‐producing taxa and increased pro‐inflammatory species, in disturbed immune signaling, T‐helper17/T‐regulatory cells imbalance, disturbed tryptophan metabolism, and disrupted integrity of the blood–brain barrier. In this review, we summarize the mechanistic and therapeutic insights from studies that have explored the gut microbiome in MS.

**Method:**

We performed a literature search in PubMed, Scopus, Web of Science, and ClinicalTrials.gov from database inception to January 2025; only English‐language articles were included, comprising human MS cohorts and preclinical experimental autoimmune encephalomyelitis models. Of these, approximately 95 human and preclinical studies fulfilled the inclusion criteria. Evidence synthesis was narrative, without meta‐analysis.

**Finding:**

There has been a consistent depletion of beneficial genera such as Faecalibacterium and Roseburia, expansion of Akkermansia muciniphila, and reduction in microbial metabolites such as butyrate, propionate, and neuroactive indole derivatives in MS patients across studies. These changes promote intestinal permeability, exaggerated pro‐inflammatory cytokine responses, and microglial activation. The therapeutic approach of restoring microbial balance includes therapies such as probiotics, prebiotics, synbiotics, fecal microbiota transplantation, and dietary interventions. Early trials have shown modest improvements in relapse rates, fatigue, immune profiles, and microbiome composition. Results across randomized studies are heterogeneous, with no significant clinical benefit in several. Pilot trials report modest reductions in relapse rate (RR ≈ 0.85) and fatigue (Cohen's *d* ≈ 0.3), but several double‑blind RCTs showed no significant benefit (*p*  >  0.05) in up to 40% of participants, highlighting variable effect sizes.

**Conclusion:**

Interventions aimed at the microbiome are promising as adjunct approaches to the treatment of MS, acting principally through the restoration of SCFAs, immune modulation, and strengthening of the gut‐brain axis. Larger, longer‐term randomized trials are required to confirm clinical efficacy, define responder phenotypes, and inform personalized microbiome‐based therapies.

## Graphical abstract

The gut microbiome in multiple sclerosis (MS) shows consistent depletion of short‐chain fatty acid (SCFA)‐producing bacteria, expansion of pro‐inflammatory species, and reduced levels of butyrate, propionate, and indole metabolites. These alterations increase intestinal permeability, disrupt immune balance, and contribute to blood–brain barrier dysfunction and neuroinflammation. Microbiome‐targeted interventions, including probiotics, prebiotics, synbiotics, fecal microbiota transplantation, and dietary strategies, aim to restore microbial balance and modulate immune and gut–brain pathways. Early clinical studies report modest improvements in relapse activity, fatigue, immune markers, and microbiome composition, although findings are variable across trials.

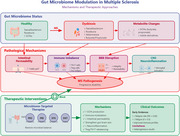

**Abbreviations**
AD: Alzheimer's Disease
ANS: Autonomic Nervous System
ASD: Autism Spectrum Disorder
BBB: Blood‐Brain Barrier
CNS: Central Nervous System
DMT: Disease‐Modifying Therapy
EAE: Experimental Autoimmune Encephalomyelitis
ENS: Enteric Nervous System
FMT: Fecal Microbiota Transplantation
GALT: Gut‐Associated Lymphoid Tissue
GLP‐1: Glucagon‐Like Peptide‐1
HPA: Hypothalamic–Pituitary–Adrenal
IBD: Inflammatory Bowel Disease
IBS: Irritable Bowel Syndrome
IFN‐γ: Interferon‐gamma
IL‐10: Interleukin‐10
MBP: Myelin Basic Protein
MOG: Myelin Oligodendrocyte Glycoprotein
MS: Multiple Sclerosis
NET: Neutrophil Extracellular Traps
PD: Parkinson's Disease
SCFA: Short‐Chain Fatty Acids
TLR: Toll‐Like Receptor

## 1 Introduction

Multiple sclerosis (MS) is a chronic autoimmune disease of the central nervous system (CNS), affecting an estimated 2.8‐3 million people globally, according to the 2023 Atlas of MS. It is characterized by various stages of nerve damage including, inflammation, demyelination, gliosis, and axonal loss. It predominantly manifests as motor dysfunction like ataxia and weakness, cognitive decline, Lhermitte sign, and sensory symptoms, as well as bowel and sexual dysfunction. These symptoms reflect the location of demyelinating lesions: inflammation of the optic nerve typically results in visual impairment; cerebellar involvement produces ataxia; spinal cord lesions give rise to bowel, bladder, and sexual dysfunction; and demyelination within cortical or subcortical regions contributes to cognitive decline. The etiology of MS includes a variety of environmental factors, including tobacco smoking, lack of vitamin D, infection by the Epstein‐Barr virus (EBV), as well as obesity and diet. This, when coupled with being in a genetically predisposed individual (e.g., those with the *HLA‐DRB1* gene polymorphism), triggers a CNS autoimmune response (Haki et al., 2024; Montgomery et al., 2024).

Over the past decade, studies have indicated the gut microbiome to be a major regulator in systemic and neuroimmune processes. It plays a crucial role in the formulation of immunological function, which is a prerequisite in the emergence of autoimmune diseases. According to various studies, the gut microbiome is proven to trigger bidirectional signaling through the gut‐brain axis, which is an intricate network of communication between the two essential components of the human body, namely, the gastrointestinal tract (GIT) and CNS (Tsogka et al., 2023; Hasaniani et al., 2024). Hence, dysbiosis, or microbial imbalance in the gut ecosystem, could be linked to a variety of neuroimmune disorders, including MS (Zhu et al., 2021). In MS, consistent alterations in gut composition have been observed, including increased Akkermansia muciniphila and *Methanobrevibacter* and decreased *Faecalibacterium prausnitzii* and *Prevotella*, forming a reproducible disease‐associated microbial signature. Experimental studies carried out on humans as well as animals suggest that the composition of gut microbiota can influence the onset, course, and severity of MS. These effects occur through microbial metabolites such as SCFAs and pathways that modulate the Th17/Treg axis and regulate blood–brain barrier (BBB) integrity (Tsogka et al., 2023; Hasaniani et al., 2024; Zhu et al., 2021).

This review provides a narrative synthesis of evidence from human and animal studies rather than a systematic review or meta‐analysis. As such, the findings should be interpreted with consideration of the methodological differences across the included literature. The review aims to summarize the current findings regarding the role of gut microbiota in MS pathophysiology and to evaluate the potential therapeutic interventions that could modulate the gut microbiome. It also highlights the need for further research to support the clinical efficacy of such interventions.

## 2 Overview of Gut Microbiome

### 2.1 Composition and Function

**FIGURE 1:**
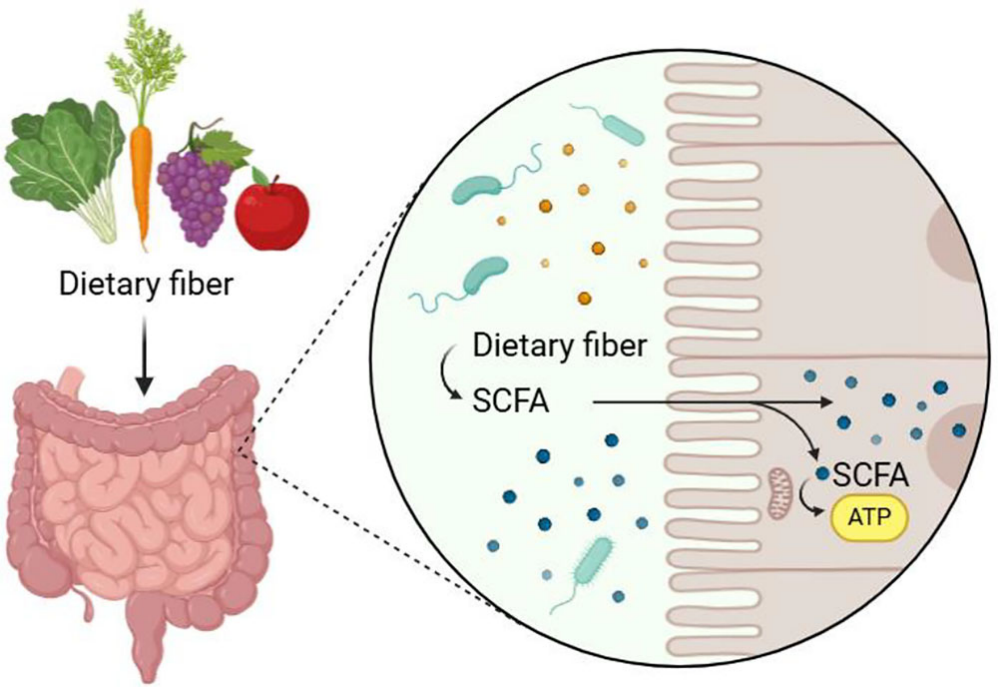
**SCFAs production by gut microbiome. ^Abbreviations:^** ^ATP, adenosine triphosphate; SCFA, short chain fatty acids.^

The human GIT harbors a diverse community of microorganisms, including bacteria, viruses, fungi, and archaea, which vary in composition by anatomical location. Collectively known as the gut microbiome, these trillions of microbes are predominantly concentrated in the colon, and their combined genetic material outnumbers human genes by more than 100‐fold. Increasingly, the gut microbiome is recognized for its crucial roles in metabolism, immune defense, and even behavior. Its composition is dynamic and influenced by factors such as age, diet, medications, and anatomical position within the GIT (Reynders, 2021). Functionally, the gut microbiota metabolizes complex carbohydrates and proteins, synthesizes essential vitamins (e.g., B and K), and produces a wide array of metabolic byproducts, such as short‐chain fatty acids (SCFAs) (**Figure** **1**), that mediate communication between epithelial and immune cells (Zhang, 2023).

At the broader taxonomic level, the healthy adult gut microbiome is dominated by the phyla Firmicutes and Bacteroidetes, which together account for the majority of bacterial species in the colon. Smaller but functionally important proportions of Actinobacteria and Proteobacteria are also consistently present. These phylum‐level patterns provide the ecological framework for understanding dysbiosis, as shifts in the relative abundance of these groups form the basis of many microbiome alterations described in autoimmune and neuroinflammatory conditions, including MS (Tsogka et al., 2023; Hasaniani et al., 2024; Zhu et al., 2021).

The stomach and duodenum have acidic and bile‐rich environments, which result in relatively low microbial populations. The dominant microbes in these areas include Lactobacillus, Streptococcus, and certain yeast species. These microorganisms play a crucial role in the early stages of digestion and offer an initial line of defense against ingested pathogens (Cao, 2024).

As we move into the jejunum and ileum, the diversity of microbes increases. Here, we find facultative anaerobes such as Streptococcus, Lactobacillus, Clostridium, Prevotella, and Fusobacterium. These organisms help with nutrient absorption and prevent pathogen colonization through a process known as competitive exclusion (Lozupone et al., 2012).

The colon, particularly the cecum and large intestine, has the highest microbial density and diversity, containing up to 10^1^ ^2^ colony‐forming units per gram of content. Dominant microbial groups include Bacteroides, Bifidobacterium, Clostridia (especially *Faecalibacterium prausnitzii* and Roseburia), Akkermansia muciniphila, methanogenic archaea, fungi, and bacteriophages. These microbes ferment non‐digestible polysaccharides, producing SCFAs such as butyrate, acetate, and propionate. These SCFAs serve as energy sources for colonocytes, regulate immune responses, and help maintain the integrity of the gut barrier (Koh et al., 2016).

For example, *F. prausnitzii* and Roseburia have shown anti‐inflammatory properties by promoting the differentiation of regulatory T cells (Tregs) and suppressing pro‐inflammatory pathways, such as the activation of nuclear factor kappa B (NF‐κB) (Machiels et al., 2014; Sokol et al., 2008). Although *Akkermansia muciniphila* degrades mucin, this process promotes compensatory mucin production by the host, thereby maintaining the integrity of the mucus layer. Additionally, its metabolites enhance the expression of epithelial tight junction proteins, collectively contributing to the preservation of intestinal barrier function (Derrien et al., 2004).

In addition to providing metabolic support, the microbiota plays a significant role in regulating host immunity by interacting with gut‐associated lymphoid tissues (GALT). This interaction influences T‐cell polarization and shapes both innate and adaptive immune responses (Belkaid and Hand, 2014). Dysbiosis, which is characterized by a decrease in beneficial commensal bacteria, an overgrowth of harmful microbes, or a reduction in microbial diversity, has been associated with various disorders, including inflammatory bowel disease (IBD), metabolic syndrome, and neuroinflammatory conditions (Peterson et al., 2008). Furthermore, early‐life microbiome colonization is crucial for immune tolerance; disruptions during infancy have been linked to increased lifetime risk of autoimmune diseases, including MS. To put these descriptions into established reference frameworks, the microbial composition reviewed here reflects the seminal findings obtained from the NIH‐funded Human Microbiome Project (HMP), which represented the first large‐scale standardized microbial genomic catalog of healthy individuals across multiple body sites (The Human Microbiome Project (HMP) Consortium 2012). Subsequent integrative analyses, including the comprehensive overview by Gilbert et al., better outline how interindividual and functional diversity shape the stability and resilience of microbial ecosystems (2016). These landmark references provide foundational context for understanding the alterations in microbiomes as observed in MS across diverse cohorts.

The aforementioned systemic and immune effects are directly relevant to MS, where altered SCFA and tryptophan derivative levels and impaired immune regulation have been implicated in neuroinflammation. Therefore, understanding the distribution and function of gut microbes in different regions of the GIT is essential for uncovering their impact on host health and disease.

### 2.2 Gut‐Brain Axis

The gut‐brain axis is a bidirectional communication network that involves gut microbes and their metabolites, the enteric nervous system (ENS), the autonomic nervous system (ANS), neuroendocrine signaling, and the CNS (**Table** **1**). Recent evidence suggests that dysbiosis plays a role in the pathogenesis of both GIT diseases such as IBD and irritable bowel syndrome (IBS) and neurological or psychiatric conditions including anxiety, depression, autism spectrum disorder (ASD), Alzheimer's disease (AD), MS, and Parkinson's disease (PD) (Cryan et al., 2019; Yoo et al., 2020).

**TABLE 1:** Pathways connecting the gut microbiome to brain (Gut‐brain axis).

Pathway	Mechanism of action	Key components	Peripheral / systemic immune effects	Impact on brain
Neural (Sorboni et al., 2022)	Direct signaling via nerves connecting gut to brain	Vagus nerve, ENS	Modulation of immune tone via vagal regulation of cytokine production	Alters stress response, mood, and cognition
Immune (Sorboni et al., 2022)	Microbial antigens and metabolites regulate immune cells	Cytokines, T cells, antigen‐presenting cells	Th17/Treg imbalance, increased IL‐17 and IFN‐γ, reduced immune tolerance	Promotes neuroinflammation and immune cell infiltration
Endocrine (Yoo et al., 2020, Sorboni et al., 2022)	Microbiota influence stress hormone release	HPA axis, cortisol	Chronic stress signaling amplifies systemic inflammation	Alters neuroendocrine regulation and neuroimmune interactions
Metabolic (Sorboni et al., 2022)	Microbial metabolites enter circulation	SCFAs, tryptophan metabolites	Reduced SCFAs impair Treg differentiation and increase systemic inflammation	BBB disruption, microglial activation
Microbial neurotransmitters (Cryan et al., 2019; Yoo et al., 2020; Sorboni et al., 2022)	Microbial modulation of neurotransmitter synthesis	Serotonin, GABA, dopamine	Neurotransmitters modulate immune cell activity and cytokine release	Affects mood, sleep, and neuroimmune signaling

**Abbreviations**: ENS, enteric nervous system; GABA, gamma‐aminobutyric acid; GLP‐1, glucagon‐like peptide‐1; HPA, hypothalamic–pituitary–adrenal; SCFAs: short‐chain fatty acids.

Communication between the gut and brain occurs through multiple pathways. The vagus nerve and spinal ANS provide direct neural signaling routes. The ENS, a complex intrinsic network within the GIT, also contributes significantly to gut‐brain communication. Specific gut bacteria have been shown to influence brain activity by stimulating vagal afferent neurons, thereby forming a direct neural bridge between the microbiota and the CNS. Studies investigating the gut‐brain axis have demonstrated a critical role for the gut microbiota in orchestrating brain development and behavior (Cryan et al., 2019; Yoo et al., 2020; Sorboni et al., 2022). The immune system is emerging as an essential regulator of these interactions (Sorboni et al., 2022).

These processes, though listed as categories, not only interact extensively but also at the level of the peripheral immune system. The regulation of neurotransmitter production and microbial downstream activity affects the differentiation of immune cells and the secretion of cytokines, and immune‐derived mediators provide feedback to the regulation of the nervous and endocrine systems. In multiple sclerosis, the activation of the peripheral immune system serves as an important link between the dysfunctioning gastrointestinal tract microbiota and the central nervous system.

### 2.3 Immune Modulation by Microbiota

Beyond its neurological influence, the gut microbiome plays a fundamental role in modulating host immunity. It interacts with both the innate and adaptive immune systems, helping maintain intestinal homeostasis and suppressing inflammation. Through the production of SCFAs and other metabolites, the microbiota promotes the development of regulatory T cells (Tregs), strengthens the intestinal barrier, and regulates immune cell signalling. Gut epithelial cells, in turn, secrete mucus and antimicrobial peptides to separate the microbiota from host tissues and maintain barrier integrity. Disruption of these interactions can lead to increased gut permeability and the proliferation of pathogenic, gram‐negative bacteria, ultimately compromising immune tolerance and increasing the risk of infection (Rooks and Garrett, 2016; iMSMS Consortium 2022).

## 3 Gut Microbiome and MS

### 3.1 Gut Microbiome Alterations in MS

Recent research established the dramatic change in the gut microbiota of individuals suffering from MS, which shows a likely pathophysiologic link between the disease and the gut. Literature described a very dramatic change in the structure of the microbiota with an increase of certain bacteria such as *Akkermansia muciniphila* and a reduction of beneficial groups such as *Faecalibacterium prausnitzii* (Jangi et al., 2016; Lin et al., 2024). More recently, meta‐analyses further identify consistent reductions in *Prevotella* spp., members of the *Clostridium* groups IV and XIVa, the *Eubacterium hallii* group, *Ruminococcus 2*, and *Lachnospira/Blautia* (of the Lachnospiraceae family), which represent important butyrate producers, whereas *Actinomyces* species and *Alistipes* species, are found to be increased in MS patients according to a consensus identified using MS cohorts (De Gennaro, 2025; Shahi, 2023; Del Negro et al., 2023). These microbiota changes have been linked to regulatory and inflammatory mechanisms; an example is that *Methanobrevibacter* has been linked to disruptions in metabolic activity in MS patients. In addition, modulation of the gut microbiota by the environment or medications, such as food, has been postulated to have significant influence on MS evolution, and regulation of gut metabolites such as SCFAs has been implicated in the modulation of inflammation. Gut microbiota alterations predispose not just to inflammatory responses but also to treatment responses, and pointing towards implicating the gut‐brain axis as a potential therapeutic target in MS (De Gennaro, 2025; Shahi, 2023; Del Negro et al., 2023).

In addition, MS disease‐modifying drugs (DMTs) have also been found to alter microbiota composition, and treated individuals were observed to be distinct in microbial networks, indicating that therapeutic maneuvers on the microbiome can modulate immune processes (Brown et al., 2021). This evidence is indicative of the dualistic function of the gut microbiome to induce and, perhaps, inhibit MS pathogenesis by modulating unique therapeutic strategies.

While EAE studies provide mechanistic insights, the growing body of human cohort studies, including that by Chen et al. (2016) and Cekanaviciute et al. (2017), validates that MS‐associated microbial dysbiosis has direct immunological consequences observable in patient‐derived samples. Landmark human cohort studies further support these findings. In a seminal case‐control study, Chen et al. (2016) were able to show that MS patients had reduced butyrate‐producing taxa and altered microbial functional pathways impacting dendritic cell maturation. Similarly, Cekanaviciute et al. (2017) demonstrated that fecal microbiota from MS patients induced a pro‐inflammatory phenotype following transplantation into germ‐free mice, providing causative evidence that MS‐associated human microbial communities modulate host immunity (Lin et al., 2024). Recent systematic reviews and meta‐analyses that integrated data across multiple MS cohorts consistently show depletion of SCFAs‐producing *Clostridiales*, *Prevotella*, and *Faecalibacterium*, with increased *Akkermansia* and *Methanobrevibacter*, reinforcing the reproducibility of these microbial signatures in humans (Tsogka et al., 2023; Rumah et al., 2013).

### 3.2 Early Evidence Implicating Microbial Products in MS

The link established by microbes in MS had been observed even before modern methods for the investigation of the current status of the microbiome were discovered. It should be noticed that the epsilon‐toxin produced by the bacterium Clostridium perfringens has been identified to cross the blood‐brain barrier, thereby reacting with myelin and oligodendrocytes. For this reason, it is established that the titer of antibodies produced against epsilon‐toxin is high in MS patients (Gil et al., 2015; Schrijver, 2001).

In parallel, it was demonstrated that bacterial peptidoglycans are also found in active lesions of MS patients. This is taken as direct histopathological proof of the presence of bacterial antigens in the CNS. Also, the presence of bacterial peptidoglycans is known to trigger the activation of innate immune receptors like NOD‐ and Toll‐like receptors. The results make it clear that bacterial antigen translocation could be an important factor in the triggering of the immune response in the CNS in MS (Thirion et al., 2023).

### 3.3 Clinical and Experimental Studies Data

#### 3.3.1 Pre‐Clinical Studies

Pre‐clinical animal models, namely experimental autoimmune encephalomyelitis (EAE), have given strong evidence for correlation between the balance of gut microbiota and neuroinflammation. Germ‐free or antibiotic‐treated mice are strongly resistant to EAE, suggesting an essential role of microbial signals in disease onset (Lin et al., 2024). Vancomycin‐treated EAE mice, for example, show an increase in immunoregulatory *Clostridium* clusters XIVa/XVIII with higher *RORγt^+^* Tregs and lower clinical scores; reconventionalization rescues the protection (Calvo‐Barreiro et al., 2021). Increased targeted intervention with 17 human‐derived *Clostridia* strains also increased severity and demyelination in EAE, boosting butyrate levels and boosting peripheral Treg responses, effects not, however, replicated by butyrate alone, indicating other metabolites or microbial interactions (Montgomery et al., 2024). Microbial manipulations with certain species further demonstrate mechanistic functions: blood‐brain barrier (BBB) colonization by *Limosilactobacillus reuteri* increases EAE as well as suppression of SCFAs‐producers, an effect reversed by high‐fiber diets that substitute SCFAs (Ghimire et al., 2025). Moreover, a diminished *Bifidobacterium* to *Akkermansia* ratio in mice was associated with worsened EAE severity, consistent with what has been observed in MS patient cohorts, and pro‐inflammatory signaling was triggered and disease was aggravated upon treatment with *Blautia wexlerae* (Gandy et al., 2019).

Last, by employing relapse‑remitting versus chronic EAE models, differing baseline gut microbiota compositions accounted for disease course, with certain taxa being more abundant in particular forms (Samson et al., 2022). Together, these studies establish a causal, modifiable link between gut microbiota and MS disease pathogenesis through SCFAs metabolism, Tregs induction, and immune–microbiota interaction.

#### 3.3.2 Clinical Studies

Data from numerous large‐scale human cohort studies exists to show significant alterations to the gut microbiota in MS patients. In the international “iMSMS” study (576 MS patients and 1152 family members who are healthy), there was a heightened abundance of *Akkermansia muciniphila*, *Ruthenibacterium lactatiformans*, and *Hungatella hathewayi*, but a lower abundance of *Faecalibacterium prausnitzii* and *Blautia* species in MS patients (Rumah et al., 2013). A case‐control “shotgun‐sequencing” study in Danish patients (*n* = 148 MS patients and 148 controls) identified 61 species‐level microbial differences that could be related to plasma values for cytokines and gene‐expression signatures in MS (Calvo‐Barreiro et al., 2021).

### 3.4 Common Microbial Patterns in MS Patients

It has been discovered that MS patients characteristically show specific patterns in the gut microbiota, which vary from the general population. Repeated changes observed are increased prevalence of specific bacteria such as *Akkermansia muciniphila* and *Methanobrevibacter*, which contribute to immune modulation and inflammation. At the same time, decreases in anti‐inflammatory bacteria like *Faecalibacterium prausnitzii* and *Butyricimonas* have also been seen that may be attributed for the pro‐inflammatory milieu seen with MS (Jangi et al., 2016; Lin et al., 2024). Recent evidence suggests differences in microbiome composition according to MS subtype. In Relapsing‐Remitting Multiple Sclerosis (RRMS), there tends to be Akkermansia enrichment and Prevotella depletion. In secondary progressive MS (SPMS), there's less microbial diversity with greater losses in SCFAs producers. In Primary Progressive Multiple Sclerosis (PPMS), there are specific differences like increased abundance of *Clostridium bolteae*, *Ruthenibacterium lactatiformans*, fewer *Lachnospiraceae*, and *Blautia* species. These differences are evident in one study (Reynders, 2021). Region, diet, and ethnicity are additional variably expressed modifiers (Zhang, 2023; Cao, 2024). In addition, it has been seen that the gut microbiota profile in MS has been associated with differences in gene expression in the dendritic cell development pathway and interferon signaling (Lin et al., 2024). Diverse microbial communities also are associated with MS DMTs in patients, indicating that therapy can reestablish microbial homeostasis to some degree (Jangi et al., 2016). Further research is needed to further define the role for these microbe alterations in MS pathogenesis and disease progression and in therapeutic intervention (Chen et al., 2016; Rumah et al., 2013). Although there are encouraging results, there are methodological issues in studying microbiome‐MS. They are largely cross‐sectional with small numbers. Clearly, there are confounding variables like diet, antibiotics, or disease‐modifying antirheumatic drugs which greatly influence microbial community diversity. Besides, there are issues related to differences in sequencing depth or platform and analysis pipelines. More longitudinal and multi‐omics analyses are thus warranted to provide clarification into mechanisms that are potentially therapeutic (Jangi et al., 2016; Lin et al., 2024; De Gennaro, 2025; Shahi, 2023; Del Negro et al., 2023; Brown et al., 2021; Chen et al., 2016; Cekanaviciute et al., 2017; Lin et al., 2024; Rumah et al., 2013).

## 4 Mechanisms Linking the Gut Microbiota and MS

### 4.1 Interaction With Immune System

There is a two‐way relationship between the gut microbiome and the gut immunity. The microbiome regulates the development and function of gut immunity, and on the other hand, the innate and adaptive immunity of the gut influences the composition of the microbiome. Depletion of SCFAs‐producing bacteria leads to lower butyrate circulation, hampering Tregs' differentiation but favoring pro‐inflammatory T helper 17 (Th17) responses. Also, elevated Akkermansia muciniphila leads to mucin depletion in the gut mucus layer, increasing microbial translocation and activation of antigen‐presenting cells. In addition, microbial metabolism of tryptophan contributes to differences in aryl‐hydrocarbon receptor (AhR) signaling pathways. In turn, these differences regulate microglial activation or neuroinflammation within the CNS (Miyauchi et al., 2020). For instance, Th17 cells are currently widely recognized as key influencers in MS pathophysiology. Increased Th17 cells in the gut have been linked to increased disease activity and altered gut microorganisms in MS. The microbiome sends specific signals that trigger myelin oligodendrocyte glycoprotein (MOG)‐specific Th17 cells in the gut. An experiment conducted on mice colonized by two strains of bacterial families from the small intestine, namely the Erysipelotrichaceae family and *Lactobacillus reuteri*, has shown them to develop worse experimental EAE in comparison to bacteria‐free mice. The *Erysipelotrichaceae* assists in enhancing the Th17 cell activity, whereas *Lactobacillus reuteri* makes proteins that possibly mimic MOGs. Thus, the combined effect of both these strains of bacteria could be involved in the development of MS (Wilson et al., 2022). Myelin basic protein (MBP)‐specific Th17 cells as well as neutrophilic infiltration of the intestine play a similar role (Sterlin et al., 2021). Neutrophils act via the Toll‐like receptor (TLR) pathway for differentiation of Th17 cells by neutrophil extracellular traps (NET) (Kadowaki et al., 2019). All these mechanisms lead us to believe that the gut is a prime site for the encephalitogenic T17 cell's differentiation, while the microbiome plays a crucial role in forming a link between them and the CNS (Miyauchi et al., 2020). Severe MS is associated with dramatically decreased responses of intestinal immunoglobulin A (IgA) (den Besten et al., 2013). C‐C chemokine receptor type 9 (CCR9^+^) memory T cells, typically regulatory in a healthy population, become pro‐inflammatory in MS, especially in SPMS (Miyauchi et al., 2020; den Besten et al., 2013). This was via the increased production of interleukin (IL)‐17A and interferon‐gamma (IFN‐γ) in such patients. Therefore, an imbalance between the commensals of the gut promotes immune dysfunction and increase the risk of MS (Miyauchi et al., 2020).

### 4.2 Role of SCFAs and Metabolites in MS

SCFAs, such as acetate, butyrate, and propionate, are produced by fermentation of dietary fibers and resistant starch by the intestinal microbiome (Montgomery et al., 2024). Certain SCFAs are known to be produced by particular bacterial strains. Butyrate is produced mainly by *Clostridium* clusters IV and XIVa, *Eubacterium*, *Ruminococcus*, and *Faecalibacterium*, whereas the Bacteroidota phylum produces acetate and propionate (Silva et al., 2020). SCFAs are an energy source for both the body and gut microbiota. They have the ability to cross the BBB, allowing widespread anti‐inflammatory effects, influencing immune and CNS function. Immunologically, they promote Treg differentiation, and interleukin‐10 (IL‐10) production and suppress pro‐inflammatory responses from T cells, macrophages, and neutrophils. They act on BBB cells, microglia, astrocytes, and oligodendrocytes, modulating neuroinflammation and barrier integrity (Ntranos et al., 2021; Jensen et al., 2021).

Studies conducted in mice have shown that those colonized with SCFAs‐producing organisms or those directly treated with SCFAs have shown to reduce the severity of disease in the EAE model of MS. Lower SCFAs levels (especially butyrate) have been observed in feces, plasma, and serum of people with MS. Reductions correlate with increased intestinal permeability, decreased Tregs, and worsening Expanded Disability Status Scale (EDSS) disability scores. SCFAs‐producing taxa (e.g., *Butyricimonas*, *Lachnospira*, *Bacteroides*) have been found to be depleted in MS patients (Silva et al., 2020). A study using a daily propionate supplement showed a positive response from MS patients who adhered to the treatment for one whole year consistently (Ntranos et al., 2021). Findings also suggest that restoring SCFAs levels in MS patients is overall beneficial in reducing relapse rates, improving disease status, and reducing progression (Silva et al., 2020; Ntranos et al., 2021). Therefore, further studies are required to determine the exact mechanism in order to utilize this concept in the therapeutic management of MS.

Tryptophan, an essential amino acid, is metabolized by the gut microbiota into indole derivatives, tryptamine, and indole‐3‐acetate. Certain protective bacterial metabolites, such as indole‐3‐lactate and indole‐3‐propionate, are found to be decreased and correlate with lower disability in pediatric cohorts. Whereas indoxyl‐sulfate and p‐cresol‐sulfate, which are neurotoxic metabolites, are observed to be increased in relapsing‐remitting MS (RRMS) (Silva et al., 2020; Jensen et al., 2021; Ghimire et al., 2022). Isoflavones, found in soy, exhibit anti‐inflammatory effects. Gut microbes like *Adlercreutzia equolifaciens* metabolize them into S‐equol, a protective estrogen‐like compound. In EAE models, isoflavone‐rich diets reduced disease severity, but this effect required equol‐producing bacteria. Isoflavone‐driven changes in gut lipopolysaccharide (LPS) synthesis also promoted anti‐inflammatory macrophage responses (Wu et al., 2023; Campagnoli et al., 2024).

Secondary bile acids, modified by gut microbiota, are reduced in MS. These metabolites act on farnesoid X receptor (FXR) and G protein‐coupled bile acid receptor 1 (GPBAR1) receptors to suppress inflammation. Tauroursodeoxycholic acid (TUDCA), a secondary bile acid, improves EAE outcomes and is safe in people with MS, suggesting a therapeutic role (Silva et al., 2020). *Akkermansia muciniphila* is enriched in MS gut microbiota, but its effects are context dependent. This microbe degrades mucin and phytate, leading to propionate and myo‐inositol production, both linked to immune modulation in MS. It produces indoles and GABA, with evidence showing increased gamma‐aminobutyric acid levels and altered microbial gene expression in EAE models colonized with *A. muciniphila*. In some studies, *A. muciniphila* promotes EAE, while in others it correlates with vitamin K production and slower MS progression (Campagnoli et al., 2024; Li, 2022).

### 4.3 Maintenance of BBB Integrity

The BBB is the border of endothelial cells that allows very selective and permeable passage, which keeps the CNS safe from systemic immune challenges and toxins. The BBB needs to stay intact for normal functioning of the CNS. In MS, increased permeability of the BBB happens as an early pathologic event before immune cells enter and demyelination occurs. New findings have suggested that a healthy gut flora can influence in maintaining a proper functioning BBB and therefore mediate its role in MS through the gut‐brain axis. Different studies have proven unhealthy gut imbalances with changed diversity and composition have also badly impacted both structure and function of the BBB (Schumacher et al., 2024; Tankou et al., 2018; Liu et al., 2022).

In germ‐free mice, lower expression of tight junction proteins (such as occludin and claudin‐5) and higher permeability of the BBB were reversed by colonization with conventional microbiota (Schumacher et al., 2024). SCFAs result from healthy metabolic interactions between bacteria and provide energy for epithelial cells (Schumacher et al., 2024). In MS, dysbiosis was found to reduce microbial‐derived SCFAs necessary for both preservation of BBB tight junctions and suppression of neuroinflammation in EAE models. Specific bacterial components can activate microglia and astrocytes indirectly through modulation of peripheral immune tone, which, in turn, further degrades the BBB, thereby facilitating the entry of autoreactive T cells into the CNS (Tankou et al., 2018; Liu et al., 2022).

The gut flora also helps with overall body inflammation, which is very important in disrupting the BBB. Dysbiosis leading to translocation of bacterial products like LPS into the bloodstream turns on toll‐like receptor pathways on endothelial lining and immune cells. This causes the body to make pro‐inflammatory cytokines like IL‐1β and tumor necrosis factor‐alpha and worsens BBB permeability (Jia et al., 2021). Therefore, balancing microbes by using probiotics, prebiotics, and changing diet adds hope for better gut and BBB health. These treatments alter specific microbe metabolites, lower systemic inflammation, and likely restore barrier integrity, all key to slowing down MS (Borody et al., 2022).

## 5 Therapeutic Interventions Targeting the Gut Microbiome

### 5.1 Probiotics, Prebiotics, and Synbiotics

Probiotics are live microbial entities that display specific effects across strains related to host immunity and metabolism. In MS patients, *Lactobacillus casei Shirota* has shown reductions in pro‐inflammatory cytokines, and *Bifidobacterium infantis* has shown increased secretion of IL‐10 and Tregs, whereas *Clostridium butyricum* has shown increased secretion of SCFAs, which work cumulatively to down‐regulate Th17‐induced inflammation in MS patients. The probiotics used in MS trials include VSL#3, which consists of a combination of Lactobacillus, Bifidobacterium, and Streptococcus strains, and LBS, an eight‐strain high‐dose blend (Silva et al., 2020; Ntranos et al., 2021; Jensen et al., 2021; Ghimire et al., 2022; Wu et al., 2023). Meta‐analyses support their role in preventing and managing gastrointestinal (GI) disorders, such as antibiotic‐associated diarrhea and IBS, although efficacy varies by strain and host factors (Sadowsky et al., 2021).

Prebiotics are nondigestible fibers, such as inulin, fructooligosaccharides (FOS), and galactooligosaccharides (GOS), that selectively stimulate the growth of beneficial gut microbes. Other prebiotic substrates like resistant starches, pectin, polyphenols, mannose, or human oligosaccharides in breast milk could further improve the growth of SCFAs‐producing microbes like *Faecalibacterium prausnitzii* or *Roseburia* species and anti‐inflammatory immune functions involving increased secretion of IL‐10 (Browne et al., 2021). They enhance mineral absorption, promote SCFAs production, and demonstrate immunomodulatory and anti‐pathogenic effects (Browne et al., 2021). While traditional strains, such as Lactobacillus, Bifidobacterium, and Saccharomyces boulardii, are widely used, newer strains, including *Roseburia* spp., *Akkermansia muciniphila*, *Faecalibacterium prausnitzii*, and *Propionibacterium* spp., are emerging for their anti‐inflammatory and mucosal protective roles. Beyond glucans and fructans, compounds like pectin, resistant starch, polyphenols, mannose, and human milk oligosaccharides are also being recognized for prebiotic potential (Sadowsky et al., 2021; Browne et al., 2021; Hui et al., 2019).

**FIGURE 2:**
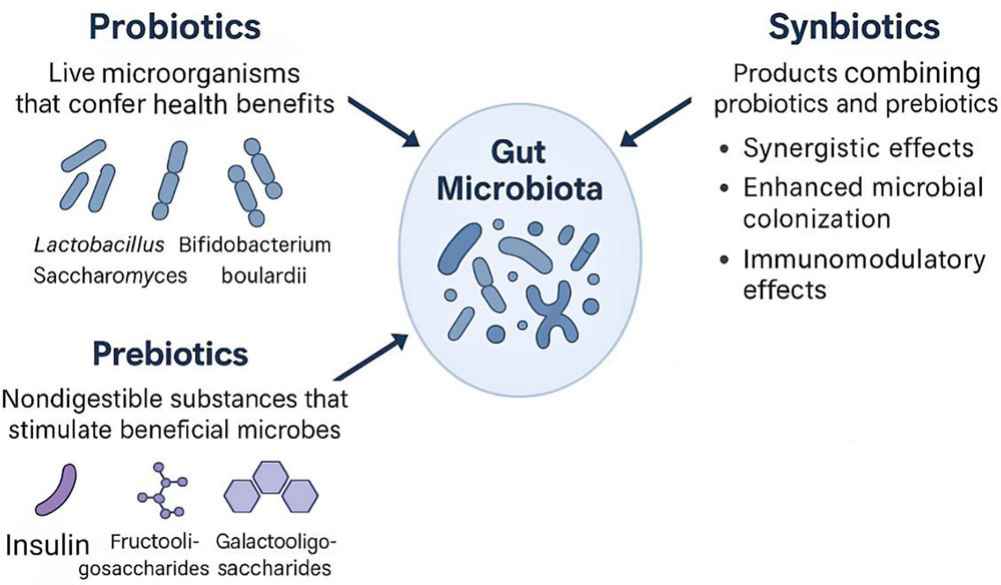
Brief description and function of probiotics, prebiotics, and synbiotics.

Synbiotics, which combine probiotics and prebiotics, offer synergistic benefits by enhancing microbial colonization, immune function, and gut homeostasis. At a mechanism level, synbiotics function through SCFAs (butyrate and propionate), improvement in gut barrier function, Treg and IL‐10 induction, and inhibition of Th17/IFN‐γ. Serum propionate has been shown to correlate with elevated frequencies of regulatory B cells and lower levels of inflammatory cytokines in MS patients (Hui et al., 2019). Both probiotics and prebiotics primarily act through their effects on gut microbiota, offering metabolic and immunological advantages (Hui et al., 2019) (**Figure** **2**).

### 5.2 Fecal Microbiota Transplantation (FMT)

Modulating the gut microbiome is now recognized as a promising therapeutic approach for various health conditions. FMT is one such approach, where stool from a healthy donor is transferred into the GIT of a patient to restore microbial balance. This technique introduces a diverse and balanced microbial consortium of bacteria, viruses, fungi, and archaea with the potential to reverse dysbiosis‐driven diseases (Hvas et al., 2019). Pilot trials in patients with RRMS show that FMT procedures are safe and that there are transient benefits to microbial composition and barrier function. The mechanisms involved are correcting microbial diversity, increasing SCFAs, fixing barrier function, and correcting immune function. These include fixing T‐reg functions and suppressing Th17. There are risks involved concerning infection transmission, donor variability, and regulatory challenges, and there are no proven benefits to neurological symptoms (Hvas et al., 2019; Zhang, 2022).

FMT has demonstrated high efficacy, particularly in treating recurrent *Clostridioides difficile* infections, with cure rates approaching 85%–90%, and is now recommended in clinical guidelines for patients with multiple relapses (Zhang, 2022; Sugihara and Kamada, 2021; Fu et al., 2022). Its use has also expanded to conditions such as inflammatory bowel disease (IBD), where some clinical trials suggest moderate improvement in symptoms (Liu et al., 2022).

In IBS, the role of FMT remains more controversial. While some randomized controlled trials have shown symptomatic improvement, others failed to demonstrate a benefit, leading to inconsistent overall findings (Cao, 2024; Straus Farber et al., 2024). Ongoing research continues to explore the mechanisms, optimal donor profiles, delivery methods, and long‐term safety of FMT in both GI and systemic diseases.

### 5.3 Dietary Modifications

**FIGURE 3:**
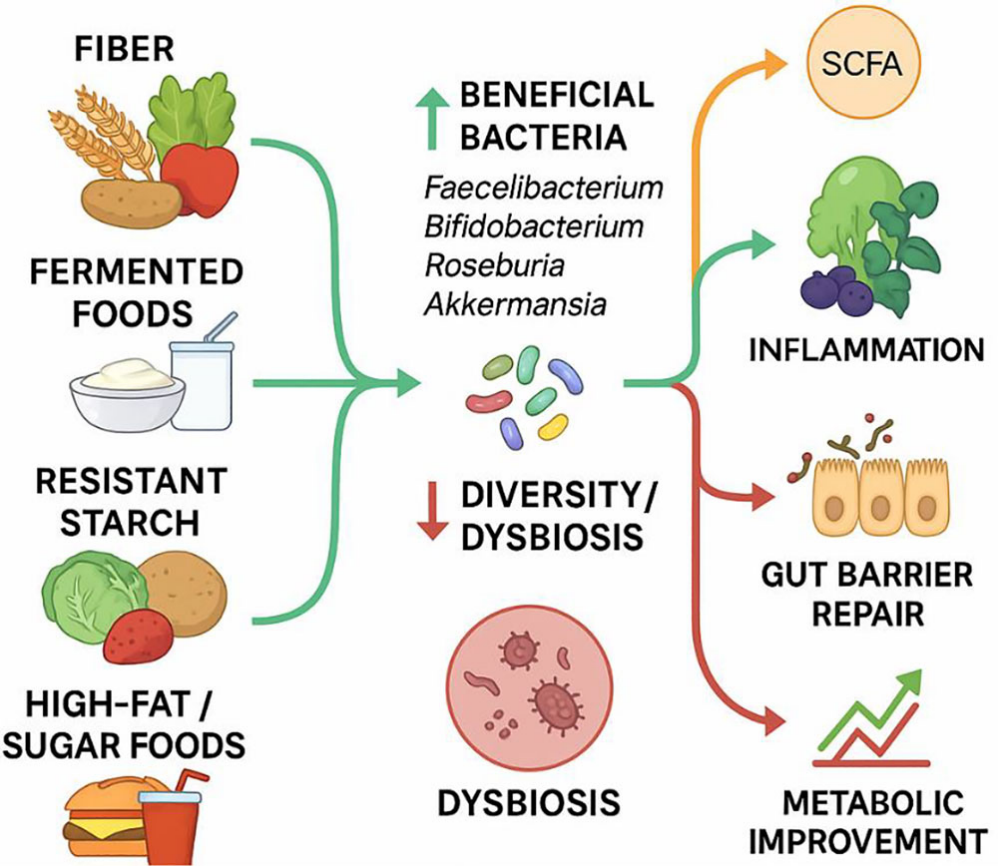
Dietary modulation of the gut microbiome.

Food components provide substrates not only for human metabolism but also for the gut microbiota, which plays an essential role in modulating immune responses involved in MS. Gut microbes ferment dietary fibers into short‐chain fatty acids (SCFAs), such as butyrate and propionate, which enhance gut barrier integrity and exert anti‐inflammatory effects. These mechanisms may help protect against the development of MS (Choi et al., 2021; Ohlsson et al., 2023; Depommier et al., 2019). Diets high in fiber and rich in whole grains, legumes, fruits, and vegetables are associated with increased microbial diversity and elevated SCFA production, which may help maintain intestinal and systemic immune homeostasis. In MS, dietary trials that have been explored are keto diets, intermittent fasting (IF) regimens, or Mediterranean/MIND diets. These diets result in increased SCFAs producers, improved microbiota diversity, elevated barrier function, increased Tregs and IL‐10, and downregulated Th17 and IFN‐γ. Pilot trials suggest that keto diets are helpful in alleviating fatigue symptoms, IF diets can modulate immune status and Mediterranean/MIND diets are helpful in establishing anti‐inflammatory microbiota (Romano et al., 2021; Duan et al., 2022; Zmora et al., 2022). Conversely, Western dietary patterns characterized by high intake of saturated fats, refined sugars, and processed foods can reduce beneficial SCFA‐producing bacteria and promote pro‐inflammatory microbial profiles, potentially exacerbating neuroinflammation (Choi et al., 2021). Functional foods, including fermented products and polyphenol‐rich fruits, have also been shown to positively influence the gut microbiome by providing live beneficial microbes and prebiotic substrates, thereby supporting anti‐inflammatory microbial activity (**Figure** **3**). (Romano et al., 2021; Duan et al., 2022; Zmora et al., 2022; Logotheti et al., 2021).

### 5.4 Microbiome Based Interventions: Clinical Trials and Experimental Therapies

Building upon the growing understanding of gut microbiota's role in MS, a wide range of microbiota‐based therapeutic approaches are under active investigation (**Table** **2**) (Blais et al., 2021; Tankou et al., 2018). These include conventional strategies such as probiotics, dietary modifications, IF, and FMT, each with varying levels of clinical validation. In a comprehensive systematic review, Tsogka et al. (2023) evaluated 13 studies involving 212 relapsing–remitting MS patients and 200 healthy controls. Their review encompassed interventions such as probiotic formulations like VSL#3 and LBS (a blend of Lactobacillus, Bifidobacterium, and Streptococcus), as well as dietary protocols, IF regimens, and case‐based reports on FMT. Mechanistically, these interventions were found to promote anti‐inflammatory cytokine profiles, modulate immune responses, and improve intestinal barrier function. Clinically, the studies reported reductions in relapse rates and EDSS scores, as well as improvements in fatigue, quality of life, and inflammatory markers, including IL‐4, IL‐6, and high‐sensitivity C‐reactive protein (hs‐CRP). Microbiome analyses further revealed reduced microbial diversity and metabolic pathway shifts favoring anti‐inflammatory functions, as observed through Kyoto Encyclopedia of Genes (KEGG) profiling. Blais et al. (2021) and Tankou et al. (2018) also reviewed human and animal studies involving strains such as VSL#3, *Lactobacillus paracasei*, *Bifidobacterium animalis*, *Escherichia coli (E. Coli)* Nissle 1917, and Prevotella histicola, supporting the therapeutic potential of commensal‐based probiotics. However, limited data on clinical endpoints and inconsistent microbiome data tempered the interpretability of their findings.

**TABLE 2:** Overview of microbiota‐based therapeutic interventions for MS: Study characteristics, interventions, mechanisms, and outcomes.

Study	Design and sample	Intervention (s)	Intervention details	Mechanism of action	Reported clinical outcomes	Microbiome/Immune changes
Tsogka et al. (2023)	Systematic review (13 studies); 212 RRMS patients, 200 controls	Probiotics (VSL#3, LBS), dietary interventions, intermittent fasting, FMT	VSL#3; LBS (Lactobacillus–Bifidobacterium–Streptococcus); intermittent fasting; dietary protocols; FMT (case reports)	↑ Anti‐inflammatory cytokine profile, intestinal barrier maintenance, immune modulation	↓ Relapse rates, ↓ EDSS, ↓ fatigue, ↑ QOL, ↓ hs‐CRP, IL‐4, IL‐6	↓ KEGG pathways; ↓ microbial diversity; cytokine modulation
Blais et al. (2021)	Systematic review (6 human, 31 animal studies)	Probiotics, commensal therapies	VSL#3; *Lactobacillus paracasei*, *Bifidobacterium animalis*, *E. coli* Nissle 1917, *Prevotella histicola*	Increases beneficial bacteria (Lactobacillus, Bifidobacterium), boosts regulatory T cells (Tregs), lowers pro‐inflammatory cytokines (e.g., TNF‐α, IL‐6), and reduces co‐stimulatory molecules on antigen‐presenting cells.	In animal studies: Increased regulatory T cells (Tregs) and reduced pro‐inflammatory cytokines, leading to clinical and neurological improvement in EAE models In human studies: Modest improvements in disability scores (EDSS) and reductions in inflammatory markers such as CRP	Enrichment of beneficial taxa (Lactobacillus, Bifidobacterium, Streptococcus), Increased microbial diversity in both models
Tankou et al. (2018a)	Pilot clinical trial	Probiotic (VSL#3)	VSL#3, unspecified dose	↓ Intermediate monocytes, ↓ CD80, ↓ HLA‐DR (immune modulation)	Decrease in pro‐inflammatory monocytes characterized by high CD14 and low CD16 expression (CD14^high CD16^low), Reduced expression of co‐stimulatory molecules CD80 and HLA‐DR on antigen‐presenting cells, Induction of a more anti‐inflammatory immune environment	↑ *Lactobacillus*, *Streptococcus*, *Bifidobacterium*
Tankou et al. (2018b)	Pilot clinical trial; 7 treated RRMS, 2 untreated, 13 controls	Probiotic (LBS)	8‐strain LBS; 3600 billion CFU/day for 2 months	↓ Pro‐inflammatory gene expression, ↓ monocytes, ↑ regulatory profile	No relapses; ↓ pro‐inflammatory markers	↑ *Lactobacillus*; ↓ *Blautia*, *Dorea*; ↓ alpha diversity in controls
Cignarella (2018)	Animal model (EAE); pilot trial in MS patients	Intermittent fasting; FMT (from fasted to EAE mice)	FMT in mice; intermittent fasting	↑ Gut flora richness, ↑ antioxidant pathways, ↓ IL‐17, ↑ Tregs	Ameliorated EAE course (in mice); altered adipokines in pilot trial	Enrichment: *Lactobacillaceae*, *Bacteroidaceae*, *Prevotellaceae*

**Abbreviations**: alpha diversity, microbial richness within a sample; CD80, cluster of differentiation 80; CFU, colony‐forming units; EAE, experimental autoimmune encephalomyelitis; EDSS, expanded disability status scale; FMT, fecal microbiota transplantation; HLA‐DR, human leukocyte antigen—DR isotype; hs‐CRP, high‐sensitivity C‐reactive protein; IL‐4, interleukin 4; IL‐6, interleukin 6; IL‐17, interleukin 17; KEGG, Kyoto Encyclopedia of Genes and Genomes pathway analysis; QOL, quality of life; RRMS, relapsing‐remitting multiple sclerosis; Tregs, regulatory T cells; VSL#3 and LBS, probiotic formulations containing *Lactobacillus*, *Bifidobacterium*, and *Streptococcus* species.

Further mechanistic insights emerged from two pilot trials conducted by Tankou et al. (2018). In the first, administration of VSL#3 in MS patients resulted in immunomodulatory effects, including reduced intermediate monocytes and downregulation of cluster of differentiation 80 (CD80) and human leukocyte antigen—DR isotype (HLA‐DR) expression, although no clinical outcomes were assessed (Cignarella et al., 2018). Microbiome profiling in this study revealed increased abundance of Lactobacillus, Streptococcus, and Bifidobacterium. In their second trial, a different group of patients received a high‐dose LBS formulation (3600 billion CFU/day for two months), which led to decreased expression of pro‐inflammatory genes, lower monocyte counts, and no relapses during the study period (Birnie et al., 2020). This intervention also resulted in enrichment of beneficial bacteria and suppression of potentially pathogenic genera such as Blautia and Dorea, alongside altered alpha diversity. In a parallel preclinical and pilot human study, Cignarella et al. (2018) and Bonnechère et al. (2023) explored the impact of IF and FMT. In EAE mice, fasting increased gut microbial richness and activated antioxidant pathways, while modulating immune responses by reducing IL‐17–producing T cells and increasing regulatory T cells. In MS patients, IF similarly shifted immune and metabolic parameters, including changes in adipokines. FMT from fasted donors conferred disease protection in the animal model, highlighting the potential for targeted microbial transfer in therapeutic contexts.

While these findings provide strong biological plausibility and early clinical signals, recent efforts have turned toward personalized and next‐generation microbiome therapies. Newer methods involve postbiotics (SCFAs or tryptophan catabolites), genetically modified probiotics expressing IL‐10 or other immunomodulators, or artificial intelligence‐based personalized microbiome therapy performed using multi‐omics analysis. These are intended to specifically address immune system dysregulation in MS patients and improve treatment outcomes, but these are still in preliminary development phases (Romano et al., 2021, Duan et al., 2022, Zmora et al., 2022). Emerging strategies aim not only to correct dysbiosis but also to precisely modulate immune responses in MS. Novel probiotic candidates such as *Akkermansia muciniphila* and *Faecalibacterium prausnitzii* have attracted attention for their anti‐inflammatory properties and ability to restore gut barrier function (Depommier et al., 2019). Engineered bacterial strains, including modified *E. coli* Nissle capable of delivering IL‐10 directly in the gut, represent an innovative approach for targeted immunoregulation (Romano et al., 2021; Duan et al., 2022). These approaches are also being studied for their potential to enhance responses to immunotherapies, not only in MS but also in broader neurological and oncological settings. Importantly, not all microbiome‐modifying interventions have demonstrated clinical benefit in MS. Indeed, several probiotic randomized placebo‐controlled trials have demonstrated no significant improvement in relapse rates, Expanded Disability Status Scale (EDSS) progression, fatigue, and inflammatory markers in up to ∼40% of MS patients (Romano et al., 2021; Zmora et al., 2022; Logotheti et al., 2021). In some studies, probiotic supplementation induced changes in the microbiome composition; this did not translate into measurable clinical benefits. Dietary intervention and IF trials have demonstrated heterogeneous responses, with individuals failing to demonstrate alterations in immune parameters or symptom burden (Romano et al., 2021; Duan et al., 2022; Zmora et al., 2022). FMT pilot studies have, by and large, been safe but have not consistently improved neurological outcomes. These neutral or negative findings underscore the presence of variability in treatment response, strain‐specific differences, and the need for rigorously designed, adequately powered RCTs. The inclusion of both positive and negative results underlines the substantial uncertainty still present in the field and avoids a potential publication bias (Logotheti et al., 2021).

## 6 Challenges and Future Directions

Most of the evidence comes from small cohorts, which restrict extrapolation of the findings to the general MS population. Small cohorts will provide high heterogeneity and bias since they might not accurately represent the heterogeneity of presentations and responses in patients (Birnie et al., 2020). Furthermore, cross‐sectional study designs utilized in gut microbiome studies render causality challenging to determine. Longitudinal designs must establish a tighter temporal relationship between microbiome alteration and MS worsening or symptom relief (Bonnechère et al., 2023).

Another challenge that is pertinent here is heterogeneity in methodology employed in the measurement of the microbiome, i.e., inconsistency in DNA sequencing methods, data analysis, and interpretation (Concato, 2013). This inconsistency hinders comparison of studies and excludes the possibility of making definitive conclusions on particular microbial communities or functions as they pertain to MS outcomes. Further, the variation in diet, medications, lifestyle, and genetic study participant population brings in layers of complexity that can complicate findings. Standardizing these, or better, controlling them in analyses, is crucial to make findings stronger (Singh et al., 2024).

Finally, most studies fail to adequately adjust for publication risk bias, wherein positive results are published more than negative or neutral results (Laidsaar‐Powell et al., 2019). In summary, although modulating the gut microbiota is likely a therapeutic modality of utility in MS, overcoming these limitations through the use of rigorous study design, large numbers of patients, and well‐standardized protocols will be very significant for future guidelines.

The intricacy of the gut microbiome's impact on MS requires personalized treatment, responding to interindividual variation in microbiota composition and host response. Recent studies highlight heterogeneity among individuals as both a challenge and a potential for precision medicine to treat MS (Shi et al., 2024; Wolter et al., 2021). The gut‐brain axis, one of the central mechanisms of MS pathophysiology, holds that bacterial metabolites could have a prominent role in neurological function, once again pointing to the significance of tailored interventions (Rooks and Garrett, 2016; Wolter et al., 2021).

Challenges to the creation of personalized therapies involve standardization of trial protocols, sample collection, and sequencing protocols that have been the source of heterogeneity of evidence on gut microbiome and MS (Zißler et al., 2024). The implementation of household pair designs in research has been promising because it minimizes variance and enhances statistical power, and thus its quality increases (Zißler et al., 2024; Cree et al., 2022).

The use of dietary interventions offers a new avenue for controlling the microbiome in a particular way. Personalized diets, probiotics, and prebiotics can be tailored to ensure a healthy gut environment, which is critical in the treatment of MS symptoms (Shi et al., 2024; Wolter et al., 2021). It has been demonstrated by certain combinations of probiotics as emerging therapeutic avenues, yet there is more that can be done to ensure strong findings (Blais et al., 2021).

Moreover, awareness of the microbial risk factors of EBV infection and microbial dysbiosis provides a new therapeutic pathway. Yet it is the translation of these understandings into clinical practice that needs to deal with the inherent complexity of individual microbiome configurations and their interaction with host determinants (Cree et al., 2022).

Briefly, although individualized approaches hold promise for MS treatment in the future by modulating gut microbiome, there are a number of challenges involved. The future will entail the integration of machine learning and artificial intelligence to efficiently harness microbiota‐associated data, which will eventually result in revolutionary and personalized therapeutic strategies (Laidsaar‐Powell et al., 2019).

Modulation of the gut microbiome as a method for the treatment of MS has attracted huge interest in terms of long‐term efficacy and safety. From the evidence presented, it can be seen that interventions like FMT have been shown to reverse or stabilize MS symptoms, for instance, through long‐term neurological improvement in patients without any adverse effects (Laeeq et al., 2023). DMTs have been shown to affect the gut‐brain axis, which has the effect of changing the gut microbiome such that it regulates immune responses in MS. Although such interventions hold potential for molding the course of the disease through the microbiome, long‐term effects on the gut microbiota structure and on patient well‐being are not known (Shahi, 2023).

Long‐term safety is of paramount importance, given the fact that MS treatment is often a question of decades. Studies demonstrate the necessity of careful long‐term monitoring, which not only seeks to track the impact of such treatments on disease activity in MS but also on the overall health and safety of patients (Correale et al., 2022). As an example, safety profiles of current DMTs based on longer trials (>5 years) highlight a requirement for balance between therapeutic and adverse effects, especially considering the dynamic nature of treatment modalities and their interactions with gut microbiota (Jakimovski et al., 2020).

Variation in response to microbiome‐directed therapies among individuals brings safety into play, thereby requiring individualized treatment regimens. Further research is required to establish what probiotic microbes or microbiota patterns are most valuable for treatment in MS patients since the influence of the gut microbiome in modulating systemic immune responses is critical in terms of disease management (Kujawa et al., 2023). As such, gut‐brain axis modulation discovery then remains to unlock therapeutic potential but also underscores the pressing need for longitudinal safety evaluation and randomized controlled trials to prove these new directions (Jangi et al., 2016; Boziki et al., 2020). Commercial probiotic products are still not widely accessible because reimbursement is limited, and availability varies. FMT also remains challenging to implement, as it depends on regulated stool banks and standardized donor‐screening procedures. Well‐defined regulatory policies and supportive economic assessments are needed to guide their fair and practical use in clinical settings.

## 7 Conclusion

This review explores the emerging role of the gut microbiome in MS pathogenesis. Induction of Treg cells and SCFA‑mediated tightening of the BBB have been associated with reductions in magnetic resonance imaging (MRI) lesion count (−12 %) and relapse frequency (−15 %) in mechanistic sub‑studies, providing a plausible bridge from microbial changes to clinical benefit. MS patients often show reduced beneficial microbes, compromised barriers, and altered immune signaling, suggesting a gut‐origin theory for MS. Therapies targeting the microbiome, such as probiotics, prebiotics, synbiotics, dietary changes, and fecal microbiota transplantation, offer promising but still limited benefits. Next‐generation strains like *Akkermansia muciniphila* and *Faecalibacterium prausnitzii* show more potential due to their anti‐inflammatory effects. Personalized and genetically engineered interventions are under investigation but require further validation. Responses differ by disease stage (RRMS  vs.  progressive), concurrent immunomodulators, and regional diet patterns; for example, probiotic efficacy was higher in treatment‑naïve RRMS patients in Western cohorts than in progressive patients on high‑efficacy DMTs. Future work should (i) integrate metagenomics, metabolomics, and immunophenotyping to define responder signatures; (ii) conduct stratified, long‑term RCTs evaluating relapse, MRI activity, and fatigue; and (iii) develop personalized, multi‑omics‑guided microbiome therapeutics.

## Author Contributions

All authors contributed to the conceptualization, drafting, and critical revision of the manuscript. All authors read and approved the final version.

## Funding

The authors have nothing to report.

## Conflicts of Interest

The authors declare no conflict of interest.

## Data Availability

The authors have nothing to report.
